# Long-Term Behavior and Durability of Alkali-Activated Clay Mortars

**DOI:** 10.3390/ma13173790

**Published:** 2020-08-27

**Authors:** Aspasia Karozou, Stavroula Konopisi, Eleni Pavlidou, Maria Stefanidou

**Affiliations:** 1 School of Civil Engineering, Dept. of Civil Engineering, Aristotle University of Thessaloniki, University Campus, 56124 Thessaloniki, Greece; konopisi@civil.auth.gr (S.K.); stefan@civil.auth.gr (M.S.); 2 Solid State Section, Physics Department, Aristotle University of Thessaloniki, Agiou Dimitriou, 54636 Thessaloniki, Greece; elpavlid@auth.gr

**Keywords:** clay mortars, activating solutions, wetting–drying, freeze–thaw cycles, microstructure

## Abstract

The need to increase the durability of clay-based materials, due to their inherent low strength and vulnerability in contact with water, led researchers to examine different options. In this paper, clay mortars were produced using four different activating solutions. Alkali hydroxides, alkali carbonates, and alkali silicates activating solutions were used. Interest is given to long term properties while their behavior to wetting–drying and freeze–thaw cycles is recorded. In total, the results of the experiments indicated the positive effect of the potassium metasilicate on mechanical characteristics presenting, however, low performance at wetting–drying. The combination of sodium metasilicate with sodium hydroxide solution has also presented a positive effect on both mechanical and physical properties. In contrast, sodium carbonate acted better in enhancing physical properties and granting water-resistant abilities. Moreover, the performance of the specimens mixed with water–glass addition presented excellent volume stability and low mass loss in durability tests.

## 1. Introduction

Low-cost materials that result from binders using activating solutions represent a sustainable option in the construction sector, not only for compatible and durable repair works but also as alternative solutions for new constructions. The “soft” nature of earthen materials is well-known, and up till now, various methods have been used to achieve stabilization, such as adding different binders, including Portland cement, fly ash, and lime [1,2]. Various issues emerge, however, such as the reduction of cement consumption in total and the reinforcement of earthen structures without the use of cement, that need to be examined to move towards sustainable building practices. The mechanism of alkaline activation of clays and soils as precursors, is under investigation in the last period, to enhance the physical-mechanical properties which are essential in determining the long-term durability of mortars [3]. As abundant materials, the development of alternative products could lead to many environmental, social, and economic advantages [4,5].

Chemical modification of earthen materials is likely to be achieved through inorganic polymerization. Specifically, alkali-activated products occur by dissolving through thermal treatment of the aluminosilicate network contained in a solid material which is called the precursor by using an alkali solution called the activator [6]. Nevertheless, the suitability of these aluminosilicate solid materials as precursors for the alkali activation process is a subject that should, in all cases, be examined before application. Usually, the preferable heating temperatures to achieve the polymerization of an aluminosilicate precursor such as clay are between 60 °C and 90 °C [7]. However, scientific studies prove that the alkali activation reaction of calcium–aluminosilicate systems can also take place in ambient conditions [6,8,9].

Moreover, different activators can be used according to the precursor in hand, thus, more suitable for earthen materials are considered the solutions of Ca(OH)_2_, NaOH, and KOH. These activators are chosen for dissolving the clay minerals efficiently while modifying the binding networks [6,10,11]. In general, the nature of the precursors and activators determine the treating conditions applied to avoid efflorescence and achieve higher stability [12,13,14].

As for alkali-activated materials, capillary absorption tests have proven that their pore networks are sufficiently helical, leading to a low capillarity [15,16]. Other researchers have found that their permeability to pure water or solutions of different ions is higher or similar than that of cement concrete [17,18].

Nevertheless, the long-term behavior and the durability of alkali-activated clay mortars in wet–dry and freeze–thaw cycles are of interest since the longevity of these materials is crucial for their utilization both in old and in modern structures and requires further studying. Additionally, the lack of applicable regulations constitutes their use more difficult. Most studies concerning such durability tests deal with alkali-activated mortars and concretes, with admixtures of industrial waste binders, such as fly ash and slag [3,19]. Moreover, the durability tests usually follow up the standards for Portland cement and concern resistance to acid attack, high temperatures, fire, and freeze–thaw [19]. The performance of such alkali-activated materials under freeze–thaw durability experiments has been proven beneficiary, presenting high resistance [19].

The frost resistance of cement depends mainly on physicomechanical rather than chemical factors, such as the porous structure [20,21]. There is a variety of reports of the performance of alkaline activated materials in the freeze–thaw process, which in some cases perform better than comparable cement concrete exposed under the same conditions [22,23].

However, there is a little reference for wet–dry cycling in alkali-activated materials, since such a test is proven not to cause a significant deterioration in cement products [3]. Furthermore, different testing methods have been reported for wetting and drying, each one being adapted to simulate different environmental conditions [3,24]. Although the specimens were exposed to different environments (temperature, relative humidity) at different ages, dimensional and/or mass measurements did not show a significant effect on materials performance [25,26,27]. However, some damage may be attributed to calcium and alkali leaching of the material with efflorescence effects [28].

This study focuses on examining the long-term effect of various alkali activators in earthen mortars. The mortars were submitted in low thermal treatment (40 °C) and ambient conditions. Since state-of-the-art research is dealing with alkali-activated materials, their long-term behavior after exposure at durability cycles is of interest. For this reason, the mechanical and physical properties of the mortars are presented.

## 2. Materials and Methods

### 2.1. Materials and Mortars Design

In total, five different compositions were cast, including the reference one (recorded as A) with no activators, used for comparison reasons. The manufactured specimens were rectangular with dimensions (40 mm × 40 mm × 160 mm) that were used in every test conducted in this study besides open porosity. The specimens for the open porosity test were approximately 40 mm × 40 mm × 50 mm). The soil used was extracted from the island of Crete and has been characterized using XRD analysis, particle size distribution, chemical analysis, and Atterberg limits. The XRD analysis indicated that the soil particles consisted of quartz, calcium aluminum hydroxide, calcite, cancrinite, and a small percentage of muscovite. Moreover, the high content of calcium oxide of 25% was reported by the results of the chemical analysis using atomic absorption technique [29]. The specific gravity of the dry material was 1.96 g/cm^3^ (ASTM-C188-95) [30], and the color characterization by Munsell charts is 5Y 7/1 light grey.

**Figure 1:**
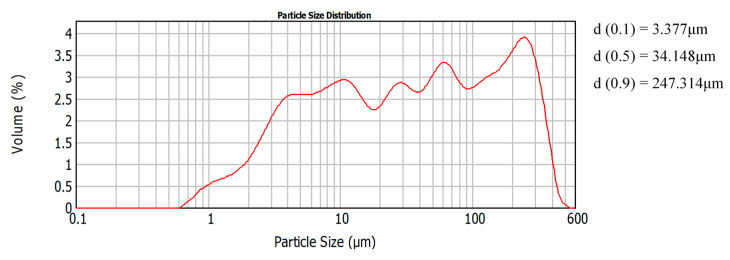
The particle size of Cretan clay used.

Moreover, the particle size distribution (Malvern 2000, Mastersizer, Thessaloniki, Greece) depicted that the particles of 2–500 μm size prevailed in the binder’s mass (Figure 1). The above analysis indicated the high volumetric percentage of silt contained in the soil (2–63 μm) of approximately 56%, with a 31% being fine sand (63–250 μm) and 13% clay particles (<2 μm). Atterberg limits were estimated (ASTM-D4318-00), while the Plasticity Index of the soil was calculated at 23.2. The liquid limit was calculated at 47.2% and the plastic limit at 24%. Thus, using the plasticity chart by Casagrande (ASTM-D2487-17) [31], the soil was classified as lean clay (CL).

The details of the mortars’ manufacture, as well as the activators used, can be found in a previous publication [29]. To clarify the acronyms PO stands for potassium metasilicate, which was used as such, SC for sodium carbonate solution, in a ratio of 70:30 water: sodium carbonate, WGS for sodium metasilicate, which was used as such, and WGN for sodium hydroxide solution mixed with water–glass at a 1:1 ratio. The water–glass used was of sodium silicate nature, with a molar ratio SiO_2_/AlK_2_O of 3.45, a pH concentration at 20 °C of 11.1, and a viscosity of 68 m·Pas at 20 °C. The activating solutions used in this work are of low viscosity (liquid form), and they are commercial products. These activators are primarily used for activation in slag, fly ash, bentonite, and pure kaolinitic clay systems [3,32,33]. However, they have not been thoroughly tested in such an impure clay system. The mortars’ composition can be seen in Table 1, while the pH values, the details of the mortar mixtures, and curing conditions can be found in a previously published research paper [29]. The workability achieved was recorded by the flow table, as mentioned in EN 1015-3.

### 2.2. Methods

In all cases, various tests were performed in both non-alkaline (A) and alkaline activated mortars (PO, SC, WGS, and WGN) of different ages (28, 90, 180, and 365 days) after manufacture. The behavior of the mortars against water penetration was examined by the conduction of capillary absorption (UNI EN15801:2010), Karsten tube penetration (EN 16302:2013), and porosity (RILEM CPC11.3) tests. Capillary absorption tests were performed at the ages of 180 days and 365 days, while water absorption through Karsten tubes was performed at 90 days and 365 days. Moreover, the open porosity tests were conducted at all ages previously mentioned, with the use of heptane instead of water [34], due to the sensitive nature of the material against direct water contact. In this study, however, the results of the 180 days and 365 days are reported.

The capillary coefficient was calculated, while immediately after the completion of the capillary test, the drying test was carried out (EN 16322:2013). The drying index (ID) was then calculated, while the results are presented as a function of time expressed in hours. The Mi-t chart represents the first drying phase of the samples, while the drying index is calculated through the integral under this curve (EN 16322:2013). Thus, the equation for the calculation of the drying index is the following (Equation (1)):

(1) $$ID=\int_{0}^{t_{f}}\frac{M_{i}dt}{M_{max}*t_{f}}\;\mathrm{where}:\;M_{i\;}=\frac{m_{i}-m_{f}}{A}$$

The symbol *m_i_* (kg) stands for the mass of the sample at a given time *t_i_* (h). Thus, m_f_ stands for the final mass of the sample recorded at the final time *t_f_* (h). Consequently, the residual amount of water of the sample at a given time *t_i_* per unit area in kg/m^2^ is symbolized as *M_i_*. Furthermore, *M_max_* is the maximum mass difference of the sample that occurs at the beginning of the test at time *t*_0_ (kg/m^2^) [35,36]. The drying index can then be calculated by Equation (1) using the simplified Equation (2) according to the European standard 16322:2013 [36]:

(2) $$ID=\frac{\sum_{i=1}^{i=n}\left[\left(t_{i}-t_{i-1}\right)*\left(\frac{M_{i-1}+M_{i}}{2}\right)\right]}{M_{max}*t_{f}}$$

Furthermore, through the Karsten tube test, the average water penetration values are calculated. The conduction of this experiment was at the ages of 90 days and 365 days. These ages were decided to have representative values both at an early age and in the long term. At the age of 28 days, the experiment was not conducted due to the vulnerable structure of the samples.

In the ages of 180 days and 365 days, the compressive and flexural strength of the mortars was examined (EN1015-11). The results recorded at the early age of 28 days and 90 days are presented in a previously published study [29]. Concerning the volume stability of the mortars, the linear shrinkage (DIN 18947:2013-08) and volume change were measured. For the latter experiment, the specimens were cured in a chamber with specific temperature and humidity conditions (23 ± 2 °C, 50% ± 5% relative humidity). The change in the dimensions and weight of the specimens was recorded daily until stabilization.

Durability tests were also carried out when the mortars reached the age of 90 days. This certain age was decided in order to allow the mortars of aerial nature (such as clay mortars) to gain strength and mass stability. Additionally, the freeze–thaw and wet–dry cycles applied were designed, taking into consideration the vulnerable nature of clay mortars and the realistic scenarios of exposing the mortars to deteriorating agents. These tests included freeze–thaw and wet–dry cycles, where the final mass loss, compressive strength values, porosity, and surface alteration through stereoscopic observation were recorded. The stereoscopic observation was conducted through a LEICA WILD M10 (Thessaloniki Greece) microscope for all mortars at the ages of 90 days and 365 days. Any surface modifications, including cracking and color alterations, as well as the roughness of the mortars, using qualitative, comparatively images under the microscope were recorded.

Furthermore, to define the modification of the inner structure, a microscopic examination by SEM (JEOL840A JSM, Thessaloniki, Greece) equipped with an EDS device was performed. Thus, the molar ratios of SiO_2_/Al2O_3_, CaO/SiO_2_, and M_2_O/Al_2_O_3_ (*M* = Na or K) at an early age and after the completion of one year were estimated indicatively.

## 3. Experimental Results

### 3.1. Physical Properties of the Mortars

The physical properties of the clay mortars were recorded and presented here after the age of 180 days to allow the carbonation mechanism to harden the soft clay structure. This decision was made after the experience recorded in previous tests [29]. The capillary absorption was measured at the 180 days and 365 days, with the capillary coefficient indicating the water absorption trend of the specimens. In all cases, the capillary coefficient was decreased through time (Table 2). The time intervals used for measuring the weight values were 0, 5, 10, 15, 30, 60, 90, 120, and 1440 min, respectively.

**Figure 2:**
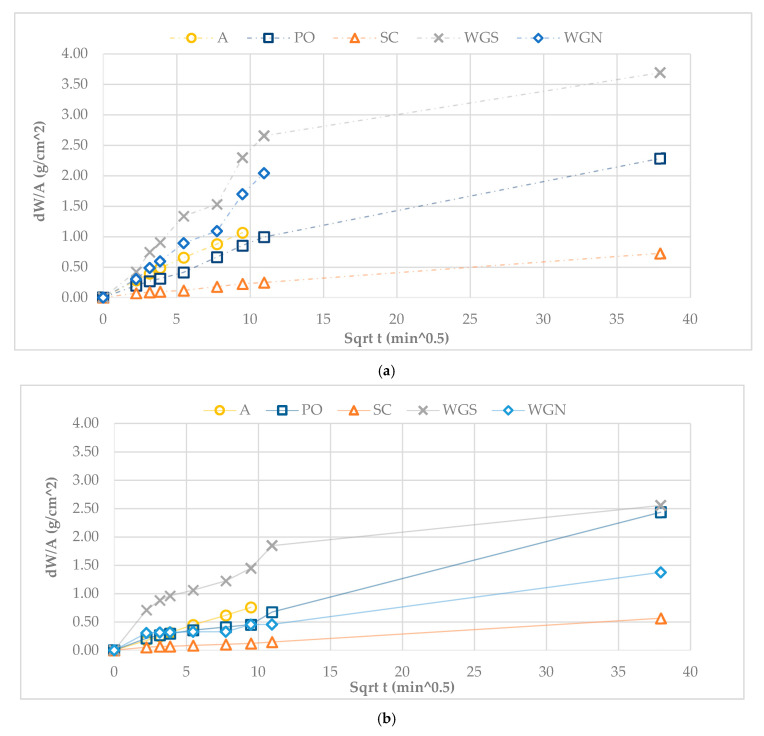
Capillary absorption of the mortars at (**a**) 180 days and (**b**) 365 days.

In general, WGS mortar presented the highest absorption rate due to capillary in the ages tested, without presenting any material loss (Figure 2). The decrease of the capillary coefficient by 20.7% at the age of 365 days indicates a more stable structure (Table 2). However, the results differ in the case of WGN mortar, since after 24 h in contact with water, the specimens suffered material loss without being able to complete the experiment at the age of 180 days. Nevertheless, the annual results showed a more stable structure, with WGN showing no material loss. Despite presenting a fast-initial absorption rate at the age of 180 days, when tested again at 365 days, the rate of absorption was reduced significantly, as can be noted by the significant difference of 63.6% between the two values of capillary coefficient (Table 2). Overall, the mortars that were activated with sodium metasilicate and sodium hydroxide solution (WGS, WGN) presented the highest absorption rate values at the age of 180 days. However, results differ at the age of 365 days for both mortars, since WGS showed the highest absorption rate, while the WGN mortar, as mentioned, had a significantly lower absorption rate. This fact is probably justified by the density of the geopolymer gel, being in the case of WGN less dense, and in the case of WGN, much denser [37], a fact justified by the porosity values as well. By the SEM analysis, in the case of WGN mortar, the loss of sodium through time (leaching effect) could have resulted in a less absorbent structure [37]. PO mortar presented low values of capillary coefficient at both ages tested, with higher final absorption value at 365 days (Figure 2). The low porosity values, as seen in Table 2 for both ages, indicate a dense formation that resulted in lower water uptake [12]. In both cases, SC mortar presented the lowest absorption rate through time, showing a 28% decrease in capillary coefficient values. Moreover, it is observed that the untreated mortar A was unable to complete the test until at all ages examined. Overall, the results come to an agreement with literature for alkali-activated metakaolin or natural pozzolan-based binders that are porous and present high capillary suction [3].

The conduction of the drying test started immediately after the completion of the capillary absorption test, as a reverse capillary test. In the case of the reference mortar A, this test was not able to be conducted since, at both ages, the samples were destroyed before completing the capillary absorption test. All other specimens were weighed using the same time intervals as the capillary absorption test and after that daily up to 960 h when all the samples have reached equilibrium with the environmental conditions (stable measurement). Weight stabilization of the specimens occurred at different times for each mortar during the total duration of the experiment. The determination of the drying curve was done after calculating the residual amount of water present in the specimen per unit area referred to as Mi (kg/m^2^). Since the drying index describes the resistance of the material to drying, it can be claimed that a low value of ID reflects an overall easier drying behavior [35,38].

In total, ID values were decreasing for all the samples tested through time, while the highest ID value was recorded for the WGN samples in the long term.

**Figure 3:**
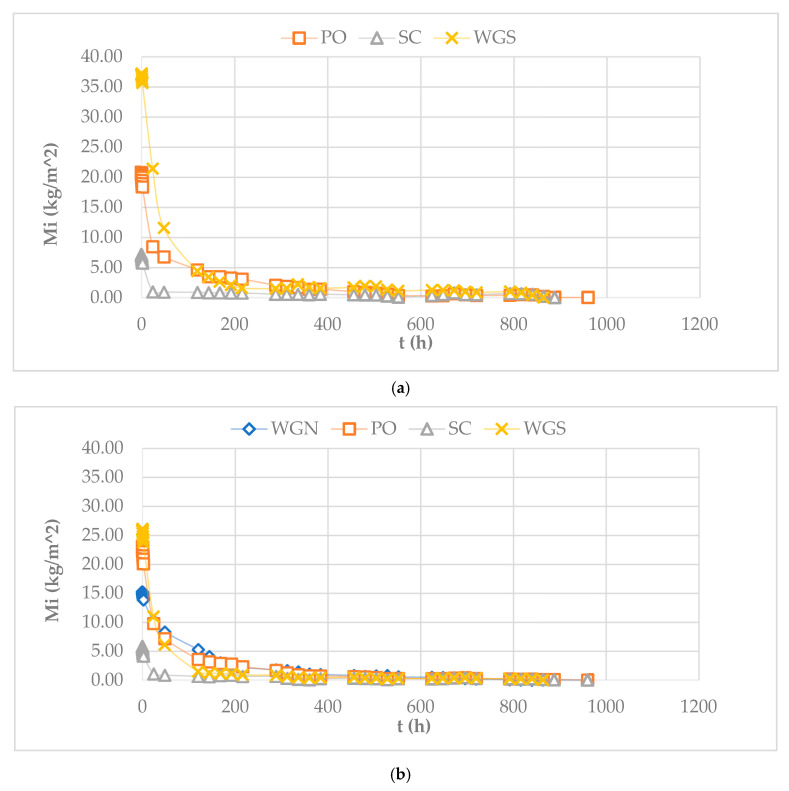
Drying curves of the mortars at (**a**) 180 days and (**b**) 365 days.

Figure 3a,b depict the drying curves of the mortars at later ages. A higher slope of the curve to the horizontal axis reflects materials with high liquid conductivity (porous materials) [36]. The final time of the drying test at 180 days was approximately the same for all the samples tested (Figure 3). Moreover, it can be observed that WGS mortars have a higher liquid conductivity compared to the other two mortars, a fact that is also justified by the high porosity values measured at both ages (Table 2, Figure 3). Presenting the lowest values of drying index at both ages tested, WGS mortars have the fastest drying behavior comparatively, with a generally distinct and long first drying phase, a fact that agrees with their high porosity values. It is also noted that WGN mortars showed the highest resistance to drying at 365 days compared to all the treated mortars tested (Table 2). Despite presenting similarly low porosity and capillary coefficient values with PO mortar at the age of 365 days, the drying behavior of the WGN mortar is significantly different, exhibiting low liquid conductivity.

Moreover, during the conduction of the experiment, efflorescence was observed on the surface of the WGN mortars. Efflorescence indicates an excess amount of unreacted sodium oxide in the pore structure that is transferred to the surface of the sample, with the presence of water through capillary. Then, the transferred alkalis react with the atmosphere, thus causing carbonation known as efflorescence [6,39]. This phenomenon that also occurred in WGN mortar shows a low exchangeability, while it can lead to a further deterioration of the system.

The PO mortars presented a low resistance to drying, with a comparably high liquid conductivity, an interesting fact considering their low porosity values (Table 2). Additionally, the second most porous mortar SC also showed a fast-drying behavior with low values of ID and a shorter first drying phase.

In Figure 3, the final drying time of the mortars can be distinguished. The mortars PO and SC presented a more extended drying period, while all mortars previously tested showed improved drying behavior with lower ID values. Moreover, despite the reduction in porosity values through time, the ID index was not negatively affected, since the decrease of the annual values for all samples, indicates a faster drying behavior meaning a quicker elimination of moisture (Figure 3, Table 2).

**Figure 4:**
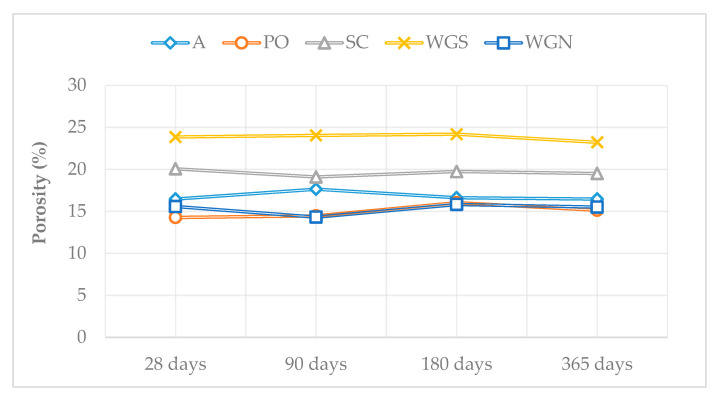
The porosity of the mortars at all ages tested.

The porosity results signify the porous structure of the WGS mortars since the porosity values were the highest recorded compared to the other mortars at all ages (Table 2, Figure 4). For PO and WGN mortars, it is noted that the porosity values remained relatively low, with the annual results being close to the values of the untreated mortar A. These values indicate the compact structure of these specimens. The high porosity values of WGS mortars, agree with the high absorption rate through capillary, while the values of the SC mortars reveal a porous structure. The high porosity values justify the low values of drying index at all ages for mortars SC and WGS.

The results of water penetration through Karsten tubes indicate the increased water absorption through time, of the most porous mortars SC and WGS. In general, it is observed that all treated mortars, besides SC, showed a higher absorption rate compared to the untreated mortar A, at all ages tested. The high tendency to water absorption of WGS and WGN mortars remains unchanged, presenting, however, a reverse behavior through time. The water absorption of WGS was increased from 90 days to 365 days by 93.2%, while the WGN mortars presented a decrease in water absorption by 53.9% (Table 3). It is noted that SC mortars showed a low water intake compared with the other treated mortars in every water absorption test conducted. PO mortars showed an average water penetration during the Karsten tube test, yet still higher than the untreated mortar A that had an overall low water intake.

**Figure 5:**
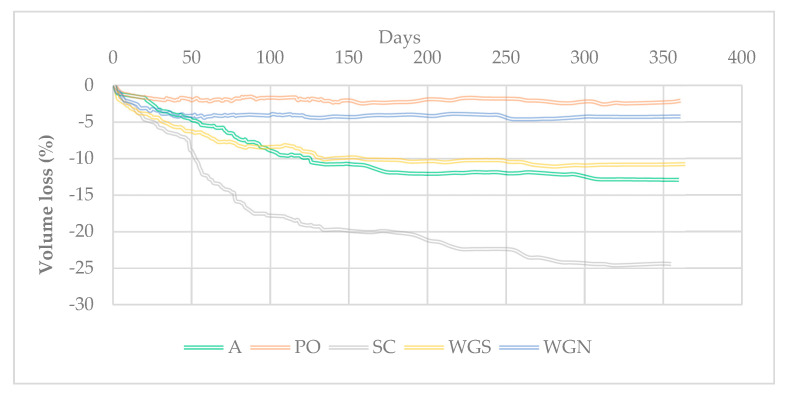
Volume loss (%) of the mortars.

Linear shrinkage and volume loss of the mortars were recorded, up until 365 days after manufacture. In Table 3, both values at 180 days and 365 days are noted as to present their progress through time (Table 3). The long-term measurements were decided to test the probable instability of the mortars through time regarding volume loss and shrinkage. According to DIN 18947, the linear shrinkage should not be more than 2% [40]. Despite the reference mortar A and mortar SC, that have barely satisfied this requirement in the long term, all the other mortars meet the standard’s requirements concerning linear shrinkage. Mortars treated with potassium metasilicate (PO) present an overall stable structure. In Figure 5, it can be detected that the percentage of volume loss through time is the lowest recorded. In general, mortars SC, WGS, and WGN presented higher values of linear and volume shrinkage, with the first showing a significant volume loss percentage in relation to the untreated mortar A, especially with the completion of one year. The total volume loss of SC mortars was 66.1% greater compared to the reference one, while PO mortars presented a 79.7% decrease in volume loss. WGS mortars presented a similar shrinkage behavior with the reference samples, showing an improvement in volume loss of 13.7%. WGN mortars presented overall good stability, with around 64% decrease in volume loss compared to A.

Overall, the mortars with the lower porosity and liquid/solid ratios presented the most stable structure also in terms of volume loss and linear shrinkage. The use of potassium metasilicate and water–glass with sodium hydroxide solution as activators have been proved beneficial in making the structure of the samples more stable.

### 3.2. Mechanical Properties of Mortars

In Table 4, the mechanical properties of the mortars are presented. The values are the average of six samples in each case. Overall, both the PO and WGN mortars presented increased values of compressive strength. An impressive 302.83% increase in one year for PO and 195.93% for WGN compared to the reference A indicate the strengthening of the structure (Table 4). In total, PO mortar presented the best performance in terms of mechanical characteristics, granting the specific activator compatible with the precursor used in this study, matching the results that derive from SEM analysis. For clay-based materials, such values of compressive strength are considered exceptionally high, since the values that are usually expected of such systems are around 1–2 MPa [41,42].

Moreover, concerning the mortar SC, it is also established in this study that sodium carbonate presents a slow strength development [32]. Even after one year, the compressive and flexural strength is considerably low, granting it as the one with the weakest behavior in terms of mechanical characteristics. The porosity values of SC also agree with the results of mechanical testing. Perhaps the slow strength development could be overcome with curing at a temperature higher than 40 °C [32] or even for an extended period, allowing a more stable and less porous structure.

The compressive test results seemed to agree with the porosity values also in the case of the WGN mortars. Notable is the fact that the WGN mortar showed the most significant compressive strength development from 180 days to 365 days by 171.19%. This fact could imply a lower strength development, especially when compared with the strong activator of potassium metasilicate. At the same time, it could also be linked to the loss of unreacted sodium through efflorescence, with an alteration of the Si/Al ratio in the structure as SEM indicates (see below). The high porosity values of WGS mortars indicate a weak performance in mechanical properties. This fact stands true for both ages, with the results at the age of 180 days being comparatively very low, while the annual values of compressive strength are comparable to the reference.

Flexural strength results presented a similar pattern, with PO and WGN mortars showing the most notable values, especially at the age of one year, with an impressive 183.33% and 39.71% increase, respectively (Table 4). In general, the mechanical characteristics are bond to the microstructure of the mortars. Through SEM analysis, it is noted that the differences in the values of Si/Al, Si/Ca, and Na, K/Al explain whether the formation of an inorganic polymeric network of alkali aluminosilicates was realized [3,43,44].

### 3.3. Durability Properties and Microscopic and Stereoscopic Observation

After reaching the age of 90 days, durability tests were carried out. These tests included freeze–thaw and wet–dry cycles, where the final percentage of mass loss, compressive strength values, porosity, and surface alteration through stereoscopic observation were recorded. The values given in Table 5 are averaged from three specimens. A full cycle in freeze–thaw durability tests consists of four hours in a chamber of −18 ± 2 °C, 10%RH, and the rest 20 h in ambient conditions (20 ± 2 °C, 65%RH). Moreover, a full wet–dry cycle includes wetting of the mortars by spraying approximately 4–5 mL of water per sample (4 cm × 4 cm × 16 cm prism) and letting them dry both in ambient conditions for three hours and then exposed them for 21 h at 40 °C. These conditions were decided based on experience due to the lack of regulations on clay-based materials. The conduction of the durability tests was until the completion of 50 cycles or until the destruction of the samples. The addition of potassium silicate in earth-based mortars proved disadvantageous concerning the wet–dry cycles since it has suffered the most significant dissolution, leading to a high amount of mass loss. Despite this deterioration, however, notable is the fact that the compressive strength value for wet–dry cycles was the highest one recorded. It is observed that both PO and WGN mortars suffered more significant mass loss than the reference mortar A regarding the wet–dry cycles, while SC mortars proved the most efficient. It is worth noting that the PO samples experienced deterioration after the completion of the 21st cycle. Thus, in Table 5, the mass change recorded is referred to as the mass of the samples after the cycle. In total, the freeze–thaw cycles showed a low mass-change effect, while the compressive strength results indicated an inadequate response to compressive strain, apart from the PO and WGN mortars. Generally, significant is the fact that despite the high deterioration of PO and WGN samples, their load-bearing capacity after the completion of the cycles was efficient.

**Figure 6:**
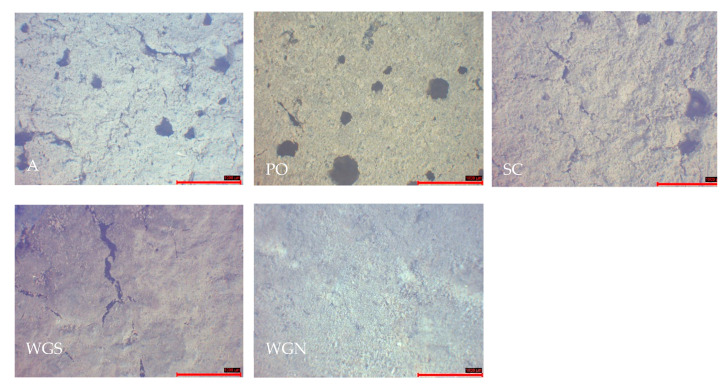
Stereoscopic observation of the mortars at the age of one year (scale 1000 μm).

The morphological characteristics of the mortars are presented at the age of one year, and after the durability cycles. The mortars examined at the age of one year showed similar characteristics with the reference sample A. Shrinkage cracks were detected, and a porous structure was evident in all surfaces observed (Figure 6). The reference mortar showed a rough, porous surface, while PO mortar presented the most compact structure and smoother surface, with few shrinkage cracks and with pores of a mean diameter 300 μm. Efflorescence was also observed microscopically on the surface of the WGN samples and inside the mass of PO mortar. Moreover, surface shrinkage cracks were detected in all mortars, with WGS mortar presenting the more significant amount. Their width was ranging from 20–110 μm, while a darker color was detected.

**Figure 7:**
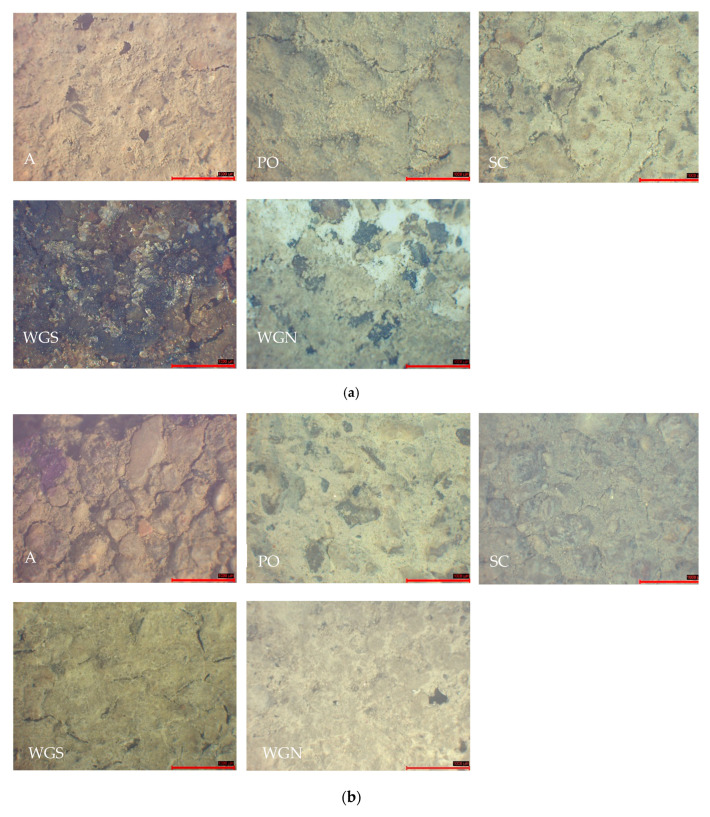
Stereoscopic observation of the mortars after the (**a**) freeze–thaw and (**b**) wet–dry cycles (scale: 1000 μm).

After the conduction of freeze–thaw cycles, all the mortars presented a more cracked and rougher surface (Figure 7a). Additionally, the surface of the mortars after the conduction of the freeze–thaw cycles showed cracking and scaling that is explained by the expansion of the inner pore water. Thus, the mass increase of most of the mortars tested is justified, since the PO, SC, and WGS presented mostly cracks and not significant scaling. For WGS mortar, the width of the cracks was between 50–160 μm after the freeze–thaw cycles. Furthermore, for all the other mortars, the range of the cracks was 40–60 μm. A disruption between the binder and the aggregates led to the mass loss of the samples WGN and A [45].

Moreover, the color alteration was apparent in WGS mortar, while in SC, some coloring spots were detected (Figure 7a). Both WGS and WGN mortars developed tarnishes and white agglomeration spots. The least porous mortar PO presented an excellent behavior in freeze–thaw cycles without showing any significant reduction in compressive strength after the completion of the cycles. The low porosity indicates a more stable mass, justifying the high values of compressive strength. The low porosity of the WGN mortar, however, is not consistent with higher strength development, presenting moderate mechanical characteristics after the freeze–thaw cycling.

The loose cohesion of the aggregates and the increase of cracks were evident after the completion of wet–dry cycles (Figure 7b). Tarnishes were again developed on the surface of the WGN mortar, while the SC mortar presented an overall good behavior against weathering. The compressive strength and porosity of the SC mortar after the wet–dry cycles are very close to the values of the annual results for the same mortar. That fact indicates the stability of the mortar against wet–dry cycles, presenting a water-resistant behavior, with almost no mass loss (Table 5). WGS mortar also presented low mass loss, yet the mechanical strength and porosity values were not improved. Significant is the fact that despite suffering mass loss, the compressive strength of the PO mortar was not significantly reduced. The results after the completion of the cycles are compared to the equivalent values of 180 days. Concerning WGN mortar, the results indicate a deterioration in mechanical strength results after the conduction of both weathering cycles. The WGN mortar suffered scaling and moderate mass loss after the wet–dry cycles, presenting, however, a very slightly improved behavior in mechanical characteristics compared to the freeze–thaw results.

**Figure 8:**
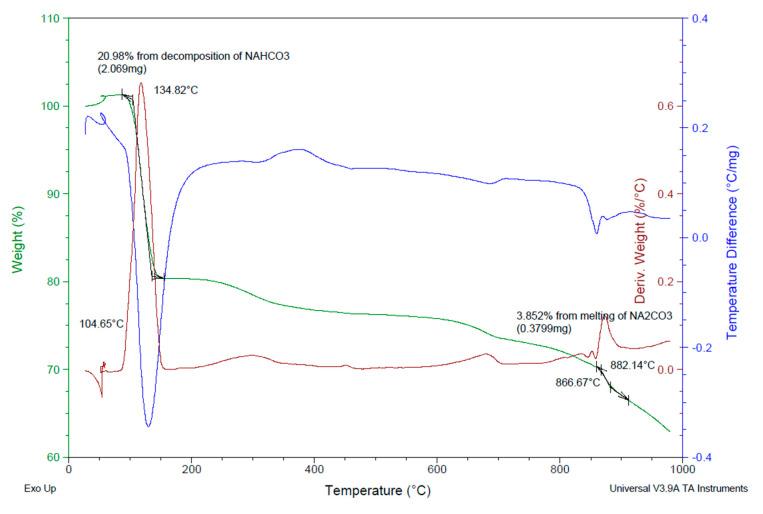
TG-DTA analysis of the efflorescence appearing on WGN mortars.

To determine the nature of the efflorescence of WGN mortars, differential thermal, and thermogravimetric analysis (DTA/TG) was performed through a TA Instruments SDT 2960 analyzer (Thessaloniki Greece) (Figure 8). The results indicated the presence of sodium hydrogen carbonate (NaHCO_3_) and sodium carbonate (Na_2_CO_3_) [46].

**Figure 9:**
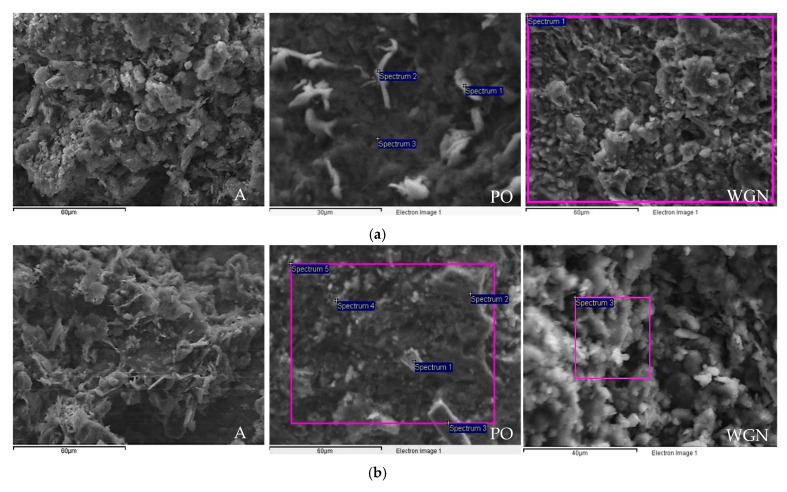
SEM images of the mortars (**a**) at an early age and (**b**) at 365 days (scale: 60 μm for A, 60 and 30 μm for PO, and 60 and 40 μm for WGN mortars).

When observing the produced mortars under SEM (Figure 9a), the reference mortar A showed a loose crystal structure at an early age, while a smoother surface was observed through time (Figure 9b). In PO mortar, rod-like crystals were detected of a potassium-based compound, with a noticeable decrease of potassium in later age (Table 6). In the case of both PO and WGN mortars, a continuous structure with small pores and few cracks was observed, also showing excellent structure cohesion. As to compare the differences in the inner structure, SEM analysis was performed at an early age (28 days) and after one year. A rougher surface with formation of leaf-like crystals was detected through SEM for WGN mortar at an early age, however, in time, a decrease of sodium content by 94% was remarked (Table 6). The presented spectrums are the average of many images, where a whole area was analyzed.

The annual results of the SEM analysis are reported, together with the results of the 28 days for comparison reasons. From Table 6, the indicative atomic ratios of the modified compositions can be calculated. The atomic ratios calculated at 28 days were Si/Al = 3.06, Si/Ca = 20.32, and K/Al = 0.73, while at 365 days the ratios were Si/Al = 4.54, Si/Ca = 14.55, and K/Al = 0.50. The increase in the mechanical properties of the PO mortar can be explained by the increase of the Si/Al ratio through time [47,48]. For the WGN synthesis, the atomic ratios at 28 days were Si/Al = 4.57, Si/Ca = 4.89, and Na/Al = 2.10, while at 365 days, the ratios were Si/Al = 3.51, Si/Ca = 5.29, and Na/Al = 0.28. These results indicate the decrease of Si/Al ratio through time by 23.16%, as well as the higher decrease of Na/Al ratio by 95.4%, facts that may explain the lower compressive strength development of the WGN mortar through time. The unbound quantity of sodium by the clay minerals in the structure probably justifies this fact. Thus, the efflorescence of WGN mortar can be explained.

## 4. Conclusions

The activation of earth materials rich in calcium was performed using different activators. Physical, mechanical, and durability properties were examined after one year. Overall, the most notable result is the high performance of potassium metasilicate (PO) in terms of mechanical properties, even after the conduction of durability tests. The achieved mechanical properties after durability tests at the age of 365 days (6.13 MPa in compressive and 3.28 MPa in flexural strength) constitute these mortars capable of different construction needs (keeping in mind their low mechanical properties as neat materials). The shrinkage trend of PO mortars was limited, judging by the low values of linear and volume shrinkage, up to the age of 365 days.

Furthermore, the improvement of their drying behavior without suffering material disintegration indicated the development of a more compact structure. The inadequate behavior of PO mortar at wet–dry cycles is a concern that should be further examined. However, the maintenance of its load-bearing capacity even after the completion of durability tests grand this mortar as a suitable building material and the specific activator very promising in order to allow further development of new advanced materials.

The water intake of sodium carbonate mortars (SC) in terms of capillary and water absorption by Karsten tubes was proven beneficial. Therefore, this water-resistant behavior of sodium carbonate shows great potential for special applications. While presenting a water-resistant behavior, the high values of porosity and, consequently, the low values of compressive strength diminish the value of sodium carbonate as an activator. Moreover, the high percentage of volume shrinkage of SC indicates an unstable structure, yet its high resistance against durability tests, especially in terms of wet–dry cycles, grand it as a promising water-resistant agent. Further study is required for the sodium carbonate as an activator that presented slow strength development and low values of compressive strength compared to the other mortars. This fact could be managed with the use of a combination of different activators or higher curing temperatures, as also literature suggests [38].

Concerning the behavior of the specimens mixed with water–glass (WGS), the results of mechanical and physical properties tested at different ages revealed a low performance. However, the fact that the specimens presented excellent stability in volume and linear shrinkage and low mass loss in durability tests, marks water–glass as a promising treating agent regarding mass stabilization.

The mechanical characteristics of the mortar with water–glass and NaOH solution (WGN), have been proven exceptional even in the long term. Therefore, this solution stands as another promising activator for clay mortars. Despite the efflorescence that was forming during the drying stage, it displayed a compact structure with low shrinkage tendency and satisfactory physical properties. Different methods of curing and perhaps a reduction in the dose of the activator are factors that could control efflorescence and should be further studied. The high tendency of these mortars to absorb water was evident during the capillary absorption test that caused the disintegration of the samples tested at a certain age.

In total, the combination of these activators with the specific earthen material to create advanced mortars proved satisfactory. Each agent has proven to act differently and enhance different properties. Further research is proposed to investigate the matter of the application of those agents at various earth-based materials and different percentages thoroughly. XRD analysis should be performed to establish the mineralogical composition of the newly formed materials. Moreover, further research on the reaction mechanism, the appropriate activators, and treatment should be withheld, to gain a better understanding of the conditions necessary to reach the optimum results.

## Figures and Tables

**Table 1:** Workability of the fresh mortars.

Mortar	Binder/Aggregate	Activator	L/S ^1^	Workability [mm]
A	1:2.5	no activator	0.65	155
PO	1:2.5	potassium metasilicate	0.62	150
SC	1:2.5	sodium carbonate solution	0.71	150
WGS	1:2.5	water–glass	0.95	155
WGN	1:2.5	water–glass with sodium hydroxide solution	0.69	155

^1^ L/S = liquid/solid.

**Table 2:** Physical properties of the mortars.

Mortar	Capillary Coefficient [g/cm^2^·min^1/2^]		Drying Index (ID)		Porosity [%]	
	180 days	365 days	180 days	365 days	180 days	365 days
A	0.120	0.082	–	–	16.63	16.47
PO	0.075	0.065	0.095	0.075	16.04	15.14
SC	0.021	0.015	0.090	0.072	19.74	19.51
WGS	0.244	0.194	0.087	0.056	24.19	23.23
WGN	0.163	0.059	–	0.123	15.83	15.51

**Table 3:** Water penetration and shrinkage of the mortars.

Mortar	Water Penetration [mL/min·cm^2^]		Linear Shrinkage [%]		Volume Loss [%]	
	90 days	365 days	180 days	365 days	180 days	365 days
A	0.053	0.035	1.48	1.92	10.82	12.93
PO	0.139	0.076	0.60	0.64	2.31	2.61
SC	0.014	0.023	1.70	1.97	20.14	21.40
WGS	0.377	0.728	1.14	1.21	10.22	11.11
WGN	0.897	0.413	1.05	1.10	4.05	4.64

**Table 4:** Mechanical properties of the mortars.

Mortar	Compressive Strength [MPa]				Flexural Strength [MPa]			
	180 days		365 days		180 days		365 days	
–	–	st.dev	–	st.dev	–	st.dev		st.dev
A	1.28	0.074	1.52	0.118	0.7	0.081	0.9	0.026
PO	5.12	0.690	6.13	0.548	1.84	0.611	2.55	0.390
SC	1.06	0.050	1.08	0.055	0.49	0.375	0.5	0.041
WGS	0.85	0.036	1.43	0.154	0.38	0.007	0.59	0.042
WGN	1.66	0.723	4.5	0.444	1.06	0.462	1.26	0.219

**Table 5:** Mass change and compressive strength of the mortars after durability tests.

Mortar	Mass Change [%]		Compressive Strength [MPa]		Porosity [%]	
	Freeze–Thaw	Wet–Dry	Freeze–Thaw	Wet–Dry	Freeze–Thaw	Wet–Dry
A	–1.16	–2.56	0.97	1.38	15.42	14.82
PO	1.34	–10.41	5.46	4.88	15.38	15.19
SC	0.11	–0.01	0.62	1.10	19.17	19.33
WGS	0.66	–0.31	0.43	0.71	23.37	24.58
WGN	–0.27	–3.27	2.25	2.41	15.24	15.13

**Table 6:** EDS spectrum analysis of (**1**) mortar PO and (**2**) mortar WGN (all results in atomic %).

(1) PO (a)							
**Spectrum (Early Age)**	**Na**	**Al**	**Si**	**K**	**Ca**	**Fe**	**O**
Spectrum 1	1.93	6.77	18.90	35.14	–1.19	1.08	36.52
Spectrum 2	4.14	7.63	22.01	23.46	2.49	0.58	39.52
Spectrum 3	6.08	10.45	32.51	1.48	0.26	0.68	48.74
PO (b)							
**Spectrum (365 days)**	**Na**	**Al**	**Si**	**K**	**Ca**	**Fe**	**O**
Spectrum 1	–	10.27	19.84	5.2	–	3.14	56.26
Spectrum 2	7.15	9.81	30.01	–	1.58	–	51.46
Spectrum 3	–	5.23	25.21	–	4.88	3.72	51.24
Spectrum 4	7.34	7.43	27.28	1.26	–	–	56.68
Spectrum 5	5.05	8.33	23.43	3.96	1.91	–	57.31
(2) WGN (a) and (b)							
**Spectrum**	**Na**	**Al**	**Si**	**K**	**Ca**	**Fe**	**O**
Spectrum 1 (early age)	9.01	4.28	19.57	1.50	4.00	1.66	59.10
Spectrum 3 (365 days)	1.54	5.57	19.49	0.83	3.70	2.25	60.83
